# Brazil’s Bolsa Família conditional cash transfer and child malnutrition: a nationwide birth cohort study

**DOI:** 10.1136/bmjgh-2024-018431

**Published:** 2025-07-20

**Authors:** Ila R Falcão, João Guilherme G Tedde, Enny Paixao, Thiago Cerqueira-Silva, Aline dos Santos Rocha, Rosemeire L Fiaccone, Natanael J Silva, Juliana Freitas de Mello e Silva, Maria Y Ichihara, Julia M Pescarini, Rita de Cássia Ribeiro-Silva, Mauricio Lima Barreto

**Affiliations:** 1 Center for Data and Knowledge Integration for Health (CIDACS), Oswaldo Cruz Foundation, Salvador, Brazil; 2 Epidemiology and Population Health, London School of Hygiene & Tropical Medicine, London, UK; 3 Department of Statistics, Federal University of Bahia, Salvador, Brazil; 4 Hospital Clinic, Barcelona Institute for Global Health, Barcelona, Spain; 5 Institute of Collective Health, Federal University of Bahia, Salvador, Bahia, Brazil; 6 School of Nutrition, Federal University of Bahia, Salvador, Brazil

**Keywords:** Global Health, Health policies and all other topics, Child health, Epidemiology, Nutrition

## Abstract

**Introduction:**

Poverty amplifies the risk of malnutrition, which is particularly harmful to children as it can perpetuate a cycle of poverty and poor health. This study aims to assess the association of a conditional cash transfer programme (*Bolsa Família* Program (BFP)) with child nutrition nationwide in Brazil.

**Methods:**

We used the Centre for Data and Knowledge Integration for Health Birth Cohort (baseline data from the National Registry for Social Programmes (CadÚnico) linked with live births and nutrition registries) to conduct a longitudinal population-based study between 2008 and 2015. This cohort study followed children from birth until 5 years old between 1 January 2008 and 31 December 2015. Children exposed were those who received the BFP benefit at any time during follow-up and were compared with those who never received it. Malnutrition outcomes were assessed using height-for-age, weight-for-height and body mass index-for-age z-scores classified according to WHO cut-offs. Binary and multinomial logistic regressions and kernel-based matching were performed. Subgroup analyses considered maternal education and urban/rural areas of residence.

**Results:**

Our cohort included 3 116 138 children born in Brazil between 2008 and 2015. BFP participation was associated with a 17% lower chance of stunting (OR 0.83; 95% CI 0.81 to 0.85). Additionally, BFP was associated with a 19% higher chance of wasting (OR 1.19; 95% CI 1.16 to 1.22). The protective association with stunting was more pronounced in children from less-educated mothers (OR 0.75; 95% CI 0.70 to 0.81) and those living in rural areas (OR 0.77; 95% CI 0.73 to 0.81). BFP participation was associated with higher overweight/obesity among children from mothers with 8 or more years of education and living in urban areas, while those with 3 or fewer years of education and living in rural areas experienced protective effects.

**Conclusion:**

Our findings suggest a complex relationship between BFP participation and child malnutrition outcomes. The study underscores BFP participation’s benefits in child nutritional outcomes, emphasising the programme’s potential to reduce stunting in all children and to reduce overweight/obesity in the most vulnerable ones. However, BFP was also associated with an increased risk of overweight/obesity, which may be a consequence of overlapping stages of Brazil’s rapid nutrition transition, a scenario that contributes to the double burden of malnutrition. Further research is needed to understand this finding better.

**WHAT IS ALREADY KNOWN ON THIS TOPIC**

- A comprehensive search in PubMed identified five relevant publications, including two recent systematic reviews, Manley *et al* 2022 and Semba *et al* 2022, which were considered the most up-to-date syntheses of the literature on the subject. The existing literature reports heterogeneous findings, with studies showing both positive and null associations between conditional cash transfer programmes (CCTs) and child malnutrition, leading to a lack of consensus on their impact. Even within Brazil, the evidence remains conflicting.

**WHAT THIS STUDY ADDS**

- Our research provides novel evidence on the association of the Bolsa Família Program (BFP), a large-scale Brazilian CCT, and child nutritional outcomes by analysing an extensive cohort of over 3 million children under five with linked data. Our large data set enabled us to apply the same approach across different subgroups, enhancing the robustness of our analyses. Unlike smaller studies, we assess differential associations across socioeconomic and geographic contexts.

**HOW THIS STUDY MIGHT AFFECT RESEARCH, PRACTICE OR POLICY**

- The study highlights the benefits that BFP participation has in child nutritional outcomes, emphasising its potential to mitigate stunting in more vulnerable populations. Additionally, our findings highlight the dual role of BFP in relation to overweight/obesity, emphasising the need for targeted policy adaptations to mitigate unintended consequences. These insights are valuable for policymakers in developing effective public health strategies to address child malnutrition and its broader socioeconomic determinants.

## Introduction

 Malnutrition, often associated with poverty and its related conditions, poses both immediate and long-term health threats, especially for young children.^1^ Despite significant global advancements in maternal and child health, malnutrition (encompassing wasting, stunting, underweight, overweight, obesity and micronutrient deficiencies) continues to afflict millions of children.^2^

Despite the decrease of 67% in chronic malnutrition (stunting) in the past two decades, it continues to exhibit an unequal distribution across the globe, with higher levels in the poorest countries.^3 4^ These disparities in child malnutrition indicators hinder progress towards achieving the 2030 Sustainable Development Goals.^2^ Although the prevalence of overweight and obesity is higher in high-income countries (7.8%) than in low-income countries (3.7%), there is a global rise in these conditions, with a particularly sharp increase in low-income and middle-income countries (LMICs).^5^,^7^ This surge is primarily attributed to shifts in dietary habits, challenges in providing and accessing healthy diets, as well as changes in physical activity and leisure patterns, exacerbated by excessive technology use^5^ and violence.^8^ This new nutrition reality has in turn contributed to the coexistence of multiple forms of malnutrition, such as the double burden of malnutrition, particularly in the most impoverished populations in LMICs.^5 6 9^

In Brazil, a country marked by social and health disparities, the prevalence of stunting and overweight/obesity among children under 5 in Brazil reached 10% and 13%, respectively, in 2020, revealing Brazil’s double burden of malnutrition.^3 4^ Elevated levels of child malnutrition emphasise the urgency of addressing poverty and reducing inequalities to improve child health, especially among those in more vulnerable socioeconomic conditions.^2 10 11^ Data from 2021 indicate that poverty affected 30% (62.9 million) of Brazilians, representing an increase of 9.6 million individuals compared with 2019.^12^

The Bolsa Família Program (BFP) (a Brazilian conditional cash transfer program (CCT)) is an example of a policy promoting the reduction of poverty and income inequality.^6^ Created in 2004, it has benefited millions of poor Brazilians, with over 14.6 million families receiving BFP grants in 2021.^13 14^ The BFP has shown positive effects on several health indicators, including reductions in child and maternal mortality.^15 16^ The BFP’s impact on child nutrition remains to be further explored.

The CCTs can improve child nutrition through direct income transfer and compliance with programme conditionalities, increasing purchasing power for food and early childhood healthcare, breastfeeding promotion, monitoring of complementary feeding and nutritional status, and increased vaccination.^617^,^20^ However, the previous studies evaluating BFP on nutritional outcomes yielded mixed conclusions about the benefits of social protection policies on child growth.^21^,^27^ Furthermore, mixed effects were observed when evaluating the combined effects of BFP coverage and municipal coverage of environmental variables (access to sanitation, water and solid waste collection) on hospitalisations and mortality due to malnutrition and diarrhoea.^28 29^ The large heterogeneity (such as analytical methods and age ranges) in the previous studies is worth noting, making it difficult to compare the results directly.^19 30^

Barriers to the effective universalisation of access to services by BFP beneficiaries may hinder the programme from fully achieving its intended role, particularly among the poorest populations. This highlights the need for specific strategies to integrate BFP with other programmes to ensure adequate access to and utilisation of health, education and social assistance services.^31^ Additionally, factors beyond the programme itself play a crucial role in reducing malnutrition, such as improved housing conditions, access to safe drinking water and basic sanitation.^18 28 29 32^

The large data set used in our study, with its rich set of explanatory variables—including housing conditions, water access and sanitation—allowed us to assess the association of BFP with child malnutrition using more robust methods and within specific subpopulations. In line with previous studies,^15 16 33^ we hypothesise that BFP association with nutritional outcomes may be more pronounced when analysed among socioeconomically disadvantaged groups.

## Methods

### Study design and data sources

This study used the Centre for Data and Knowledge Integration for Health (CIDACS) Birth Cohort,^34^ an open cohort built from linked records from the Unified Register for Social Programs (Cadastro Unico para Programas Sociais (CadÚnico)) and the live births from the Live Birth Information System (Sistema de Informacao de Nascidos Vivos) from 1 January 2001 onwards. The cohort provides detailed socioeconomic data at the individual, family and household levels, as well as information on participation in the BFP.^34^ Compared with the general population of live births in Brazil, children in this cohort are more likely to be born from younger, unmarried mothers with lower educational attainment.^34^

Additionally, for this study, the CIDACS Birth Cohort was linked to nutritional information system (Sistema de Vigilância Alimentar e Nutricional (SISVAN)) data, which includes routine measurements of children’s weight and length/height collected by primary healthcare professionals. The SISVAN databases consist of records for nutritional and food monitoring, and their quality has improved over time.^35^ This improvement is reflected in the high completeness of key variables, such as date of birth, weight and height, as well as a reduction in implausible z-scores to below 1%.^35^ Additionally, coverage has expanded each year, reaching approximately 45.4% of children under 5 in 2017.^34^

The linkage between the different databases was done using a linkage tool (CIDACS-RL)^36 37^ developed in-house (see online supplemental 1, subsection 1, efigure 1 for detailed linkage strategy and linkage accuracy).

### Study population

**Figure 1:**
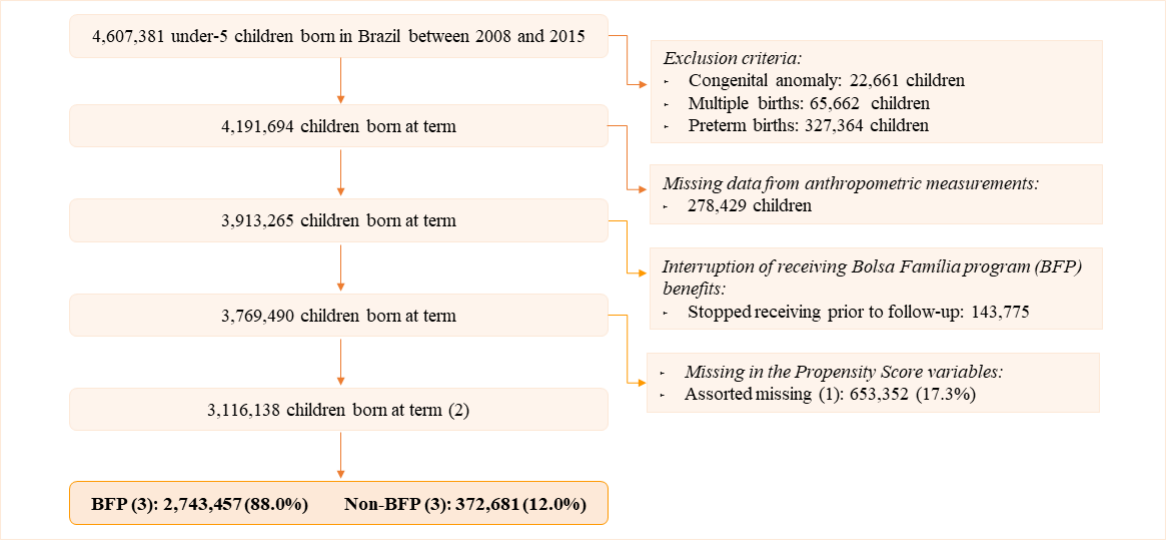
Study population to evaluate the association of Bolsa Família on nutritional indicators of under-5 children born in Brazil between 2008 and 2015. Assorted missing: this refers to missing data on any variable (but not all) used for propensity score estimation. For example, an individual may have missing data on one or more variables, but not on all variables. Only the last observation for children (one observation per child). Bolsa Família Program (BFP): received BFP benefits until the last follow-up. Non-BFP: did not receive any benefits until the child’s last follow-up date.

The eligible study population included live births from the CIDACS Birth Cohort born between 1 January 2008 and 30 December 2015, who had at least one record in the SISVAN database between 2 January 2008 and 31 December 2015 (see figure 1). This selection period (2008–2015) was based on the availability of anthropometric data (SISVAN: 2008–2018) and Bolsa Família receipt records (Cidacs Birth Cohort: 2004–2015). The study population represents only the poorest population in Brazil, specifically those eligible for social programmes.

We excluded live births born to multiple gestation, newborns with congenital anomalies and preterm births (since these conditions are associated with adverse nutritional indicators and have specific growth curves). We also excluded children with biologically implausible z-scores according to the WHO cut-offs for length/height-for-age (L/HAZ<−6 and >6), for weight-for-length/height z-scores (WHZ<−5 and >5) and body mass index-for-age z-score (BMIZ<−5 and >5).^38^ Children with inconsistencies in anthropometric data (negative height), sex and age were also excluded from the study (online supplemental 1, subsection 2, eFigure 2). BFP beneficiaries with an end date for receiving benefits before the date of the last follow-up were excluded from the primary analysis.

### Exposure

BFP is a conditional cash transfer initiative aimed at assisting poor and extremely poor families registered in the Unified Register for Social Programs (CadÚnico). Eligibility is determined based on per capita income and family composition, particularly the presence of children. During 2007–2008, extreme poverty was defined as a per capita income of ≤R$60 per month, increasing to ≤R$70 per month from 2009 to 2014. Poverty was defined as ≤R$120 per capita/month in 2007–2008, and ≤R$140 per capita/month in 2009–2014, with eligibility further contingent on having at least one child under 18 years of age or a pregnant or breastfeeding woman in the household.

Children were classified as exposed to BFP if their families began receiving benefits at any point during the follow-up period without any interruptions. Children who did not receive BFP benefits at any time during the follow-up were categorised as unexposed (see online supplemental 1, subsection 3 for detailed information on the eligibility criteria and characteristics of the BFP).

### Outcomes

Nutritional indicators for children were assessed using the last anthropometric measurements available during the follow-up period (from birth to 59 months), and z-scores were estimated based on WHO Child Growth Standards.^38^ L/HAZ categories included stunted (L/HAZ<−2) and not stunted (L/HAZ≥−2). WHZ categories were wasted (WHZ<−2), overweight risk (1<WHZ≤2), overweight/obesity (WHZ>2) and adequate (reference category) (−2≤WHZ≤1). BMIZ categories included thinness (BMIZ<−2), overweight risk (1<BMIZ≤2), overweight/obesity (BMIZ>2) and adequate (reference category) (−2≤BMIZ≤1).

### Statistical analysis

The analyses were performed following a previously published research protocol.^39^ First, the propensity score (PS) of receiving BFP was estimated using logistic regression with the following maternal variables: region of residence, level of education, marital status, electrical energy, water supply, sanitation system, waste collection, household density (overcrowding), year of entry and self-reported maternal race (ie, Asian, black, indigenous, ‘Parda’ or white; ‘Parda’ refers to individuals with predominantly black ancestry or those with multiracial or multiethnic ancestry) (see online supplemental 1, subsection 4, eTable 1, efigure 3, eTable 2 and 3 for detailed information about the PS and the descriptive analysis of the exposed and unexposed groups). The PS estimation was performed using complete data (main analysis), which assumes data are missing at random. Additionally, to test if the results were robust enough to assume that missing variables were not at random, we performed the PS estimation by incorporating a missing data category in the PS estimation. The descriptive analysis based on missing data is available in online supplemental 1, subsection 5, eTable 4–6 for detailed information about the missing data.

To estimate the effects of BFP, we used weighted logistic regression. PS weights were estimated by using kernel-based matching. Kernel matching is a non-parametric matching estimator that uses the weighted averages of almost all of the individuals in the control group to compile the counterfactual outcome.^40^ In addition, we also calculated the effects of BFP, further adjusting for confounding variables (ie, the mother’s age at birth, sex assigned at birth, low birth weight and type of delivery) (crude models and non-weighted models in online supplemental 1, subsection 6, eTable 7). All analyses were performed using Stata V.16 (Stata Corp LLC, College Station, Texas, USA), and the study was reported according to the REporting of Studies Conducted using Observational Routinely-collected Health Data statement (online supplemental 2).^41^

### Subgroup analysis

Given the well-established relationship between socioeconomic disadvantage and child nutrition,^3 42^ we conducted subgroup analyses of the effects of BFP on nutritional outcomes by maternal education (≥8 years of education; 4–7 years; ≤3 years) and residence of the mother (rural vs urban areas). For each analysis, PS was estimated separately for each population subgroup, with the variable defining the subgroup excluded from the calculation. Similarly, kernel-weighted logistic models were estimated overall and separately, within each subgroup.

### Robustness check

First, to test if our definition of exposure to BFP was robust, we used an alternative approach for selecting exposed individuals who changed their beneficiary status between two follow-ups (online supplemental 1, subsection 7). To perform this analysis, we selected the first and last follow-up for each child from birth to 59 months (online supplemental 1, eFigure 4 and eTable 8).

Second, we examined the association of BFP with nutritional indicators among primiparous women based on the variable number of previous live births (zero previous children, mothers classified as primiparous) (online supplemental file 1, subsection 8, eTable 9). Women who enrolled in BFP after their first child’s birth may be more similar to those who never received the benefit because of the programme’s eligibility criteria. The variable related to stillbirths and abortions was not used due to missing data exceeding 10%.

Third, to strengthen the validity of the findings, we assessed the effects of BFP on nutritional outcomes as continuous variables. The analytical steps (PS estimation, kernel matching and weighted linear regression) were conducted (online supplemental 1, subsection 9, eTable 10).

### Patient and public involvement

This study was based on routinely collected electronic data, without personally identifiable information, and did not require direct contact with participants. Patients and members of the public were not involved in the design, conduct or analysis of this study.

## Results

After exclusions, 3 116 138 children were included in our study, 88.0% of whom were beneficiaries of BFP. The prevalence of stunting in the population was 7.7%, and overweight/obesity was around 12%, with little variation by BFP status. In contrast, the prevalence of wasting (7.1% vs 4.4% in non-BFP children) and thinness (7.4% vs 4.8% in non-BFP children) was higher among children receiving BFP benefits (table 1).

**Table 1:** Description of the children born in Brazil between 2008 and 2015, in accordance with the receipt of BFP

Variables	BFP	Non-BFP	Total
Child’s age in months			
Mean (SD)	39.8 (15.0)	27.0 (18.0)	38.7 (15.7)
Median (IRQ)	42.8 (28.4–53.0)	24.8 (10.9–42.4)	41.8 (26.6–52.5)
Child’s age categorised, (months), n (%)			
<12	143 805 (5.1)	74 121 (27.4)	217 926 (7.0)
12–23	392 458 (13.8)	57 774 (21.3)	450 232 (14.5)
24–35	513 200 (18.0)	48 770 (18.0)	561 970 (18.0)
36–47	706 941 (24.9)	41 806 (15.4)	748 747 (24.0)
48–59	1 088 981 (38.3)	48 282 (17.8)	1 137 263 (36.5)
Child’s sex assigned at birth, n (%)			
Male	1 411 997 (49.6)	139 746 (51.6)	1 551 743 (49.8)
Female	1 433 388 (50.4)	131 007 (48.4)	1 564 395 (50.2)
Race, n (%)			
White	875 480 (30.8)	136 990 (50.6)	1 012 470 (32.5)
Black	108 092 (3.8)	8012 (3.0)	116 104 (3.7)
Asian	10 834 (0.4)	1143 (0.4)	11 977 (0.4)
Parda	1 831 534 (64.4)	124 098 (45.8)	1 955 632 (62.8)
Indigenous	19 445 (0.7)	510 (0.19)	19 955 (0.6)
Area of residence, n (%)			
Urban area	2 127 456 (74.8)	229 115 (84.6)	2 356 571 (75.6)
Rural area	717 929 (25.2)	41 638 (15.4)	759 567 (24.4)
Mother’s years of study, n (%)			
8 or more	1 430 586 (50.3)	184 771 (68.2)	1 615 357 (51.8)
4–7	1 075 025 (37.8)	73 101 (27.0)	1 148 126 (36.8)
3 or less	339 774 (11.9)	12 881 (4.8)	352 655 (11.3)
LAZ/HAZ			
Average (SD)	0.0 (1.6)	0.0 (1.5)	0.0 (1.6)
Median (IRQ)	−0.1 (−1.0 to 0.8)	−0.1 (−0.9 to 0.8)	−0.1 (−1.0 to 0.8)
WHZ			
Average (SD)	0.2 (1.2)	0.2 (1.2)	0.2 (1.2)
Median (IRQ)	0.1 (−0.6 to 0.9)	0.2 (−0.5 to 1.0)	0.1 (−0.6 to 0.9)
BMIZ			
Average (SD)	0.27 (1.59)	0.38 (1.44)	0.28 (1.58)
Median (IRQ)	0.30 (−0.63 to 1.24)	0.39 (−0.48 to 1.26)	0.31 (−0.62 to 1.24)
Categorised LAZ/HAZ, n (%)			
Appropriated (LAZ/HAZ≥−2)	2 626 875 (92.3)	250 171 (92.4)	2 877 046 (92.3)
Stunting (LAZ/HAZ<−2)	218 510 (7.7)	20 582 (7.6)	239 092 (7.7)
Categorised WHZ, n (%)			
Eutrophic (−2≤WHZ≤1)	1 826 691 (64.2)	173 935 (64.2)	2 000 626 (64.2)
Wasting (WHZ<−2)	202 339 (7.1)	11 893 (4.4)	214 232 (6.9)
Overweight risk (1<WHZ≤2)	497 208 (17.5)	54 038 (20.0)	551 246 (17.7)
Overweight/obesity (WHZ>2)	319 147 (11.2)	30 887 (11.4)	350 034 (11.2)
Categorised BMIZ, n (%)			
Eutrophic (−2≤BMIZ≤1)	1 774 057 (62.3)	172 552 (63.7)	1 946 609 (62.5)
Thinness (BMIZ<−2)	211 664 (7.4)	13 033 (4.8)	224 697 (7.2)
Overweight risk (1<BMIZ≤2)	510 842 (18.0)	53 476 (19.8)	564 318 (18.1)
Overweight/obesity (BMIZ>2)	348 822 (12.3)	31 692 (11.7)	380 514 (12.2)

BFP, Bolsa Família Program; BMIZ, categorised z-score for body mass index-for-age; LAZ/HAZ, categorised z-score for length/height-for-age; WHZ, categorised z-score for weight-for-length/height.

**Figure 2:**
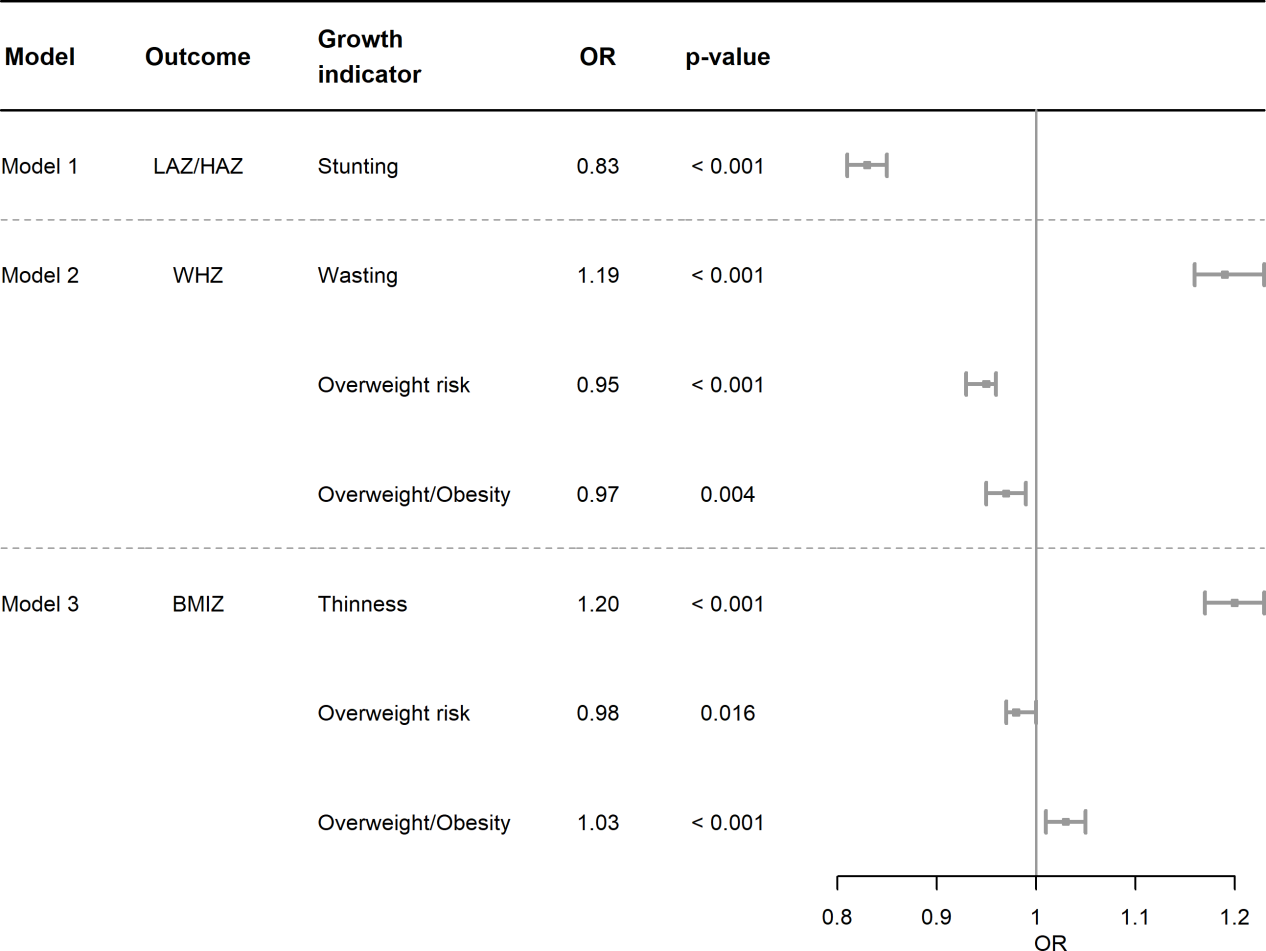
Coefficients of adjusted kernel-weighted binary (1) and multinomial (2) regressions of Bolsa Família beneficiaries on nutritional indicators (3) of children born in Brazil between 2008 and 2015. For stunting: in logistic regression results, the analysis was kernel weighted and adjusted for mother’s age at birth, sex assigned at birth, low birth weight and type of delivery. For all other outcomes (except stunting): in multinomial regression results, the analysis was kernel weighted and adjusted for mother’s age at birth, sex assigned at birth, low birth weight and type of delivery. BMIZ, categorised z-score for body mass index-for-age; LAZ/HAZ, categorised z-score for length/height-for-age; WHZ, categorised z-score for weight-for-length/height.

BFP was associated with a 17% lower chance of stunting (OR 0.83; 95% CI 0.81 to 0.85) (figure 2, eTable 7). Conversely, beneficiary children also had a 19% higher chance of wasting (OR 1.19; 95% CI 1.16 to 1.22). The association between BFP and overweight/obesity behaved oppositely according to the WHZ (OR 0.97; 95% CI 0.96 to 0.99) and BMIZ (OR 1.03; 95% CI 1.01 to 1.05) indicators.

In the subgroup analysis (table 2) by mother’s education level, BFP participation was associated with a reduction in the likelihood of stunting across all education strata. The strongest reduction was observed among mothers with fewer years of education (OR 0.75; 95% CI 0.70 to 0.81). A similar pattern was observed for overweight/obesity, with approximately a 10% reduction in the likelihood of being overweight/obese among children of mothers with fewer years of education who received BFP benefits. Conversely, BFP participation was associated with higher odds of overweight/obesity among children of more educated mothers (BMIZ—OR 1.09; 95% CI 1.06 to 1.11). BFP participation was associated with higher chances of wasting and thinness, with lower chances across subgroups with fewer educational levels (wasting—OR 1.11; 95% CI 1.01 to 1.22; thinness: OR 1.12; 95% CI 1.01 to 1.22).

**Table 2:** Coefficients of adjusted kernel-weighted binary (1) and multinomial (2) regressions of Bolsa Família beneficiaries on nutritional indicators of children born in Brazil between 2008 and 2015, in accordance with subgroup analysis

Subgroup models/outcomes (3)	LAZ/HAZ	WHZ			BMIZ		
	Stunting (LAZ/HAZ <−2)	Wasting(WHZ< −2)	Overweight risk (1<WHZ≤2)	Overweight/obesity (WHZ>2)	Thinness(BMIZ< −2)	Overweight risk (1<BMIZ≤2)	Overweight/obesity (BMIZ>2)
	**OR(95% CI)**	**OR(95% CI)**	**OR(95% CI)**	**OR(95% CI)**	**OR(95% CI)**	**OR(95% CI)**	**OR(95% CI)**
Area of residence							
Model 1: urban (n=2 350 712)	0.86***(0.84 to 0.88)	1.24***(1.20 to 1.28)	0.95***(0.94 to 0.97)	1.00(0.98 to 1.02)	1.25***(1.21 to 1.29)	1.00(0.98 to 1.01)	1.07***(1.05 to 1.10)
Model 2: rural area (n=7 54 882)	0.77***(0.73 to 0.81)	1.08**(1.03 to 1.15)	0.92***(0.89 to 0.95)	0.88***(0.85 to 0.92)	1.08**(1.02 to 1.14)	0.94**(0.91 to 0.98)	0.91***(0.87 to 0.95)
Mother’s education (in years)							
Model 1: 8 or more (n=1 612 175)	0.87***(0.85 to 0.90)	1.25***(1.21 to 1.29)	0.96***(0.94 to 0.98)	1.02(0.99 to 1.04)	1.26***(1.22 to 1.30)	1.00(0.98 to 1.02)	1.09***(1.06 to 1.11)
Model 2: 4–7 (n=1 144 075)	0.81***(0.78 to 0.84)	1.15***(1.10 to 1.20)	0.94***(0.92 to 0.97)	0.95**(0.91 to 0.98)	1.14***(1.09 to 1.19)	0.97*(0.95 to 1.00)	1.00(0.97 to 1.03)
Model 3: 3 or less (n=3 49 625)	0.75***(0.70 to 0.81)	1.11*(1.01 to 1.22)	0.90**(0.84 to 0.95)	0.86***(0.80 to 0.93)	1.12*(1.01 to 1.22)	0.91**(0.85 to 0.97)	0.88**(0.82 to 0.95)

*p<0.05; **p<0.01; ***p<0.001.

(1) Logistic regression results: analysis weighted and adjusted for sex assigned at birth, mother's age at birth, low birth weight and type of delivery. (2) Multinomial regression results: analysis weighted and adjusted for sex assigned at birth, mother's age at birth, low birth weight and type of delivery. (3) LAZ/HAZ, WAZ, WHZ and BMIZ.

BMIZ, categorised z-score for body mass index-for-age; LAZ/HAZ, categorised z-score for length/height-for-age; WAZ, categorised z-score for weight-for-age; WHZ, categorised z-score for weight-for-length/height.

In the subgroup analysis by area of residence (table 2), BFP beneficiary children living in rural areas generally experienced a greater protective effect. For stunting, BFP participation was associated with a 23% lower likelihood (OR 0.77; 95% CI 0.73 to 0.81). However, even in rural areas, BFP participation was linked to higher odds of wasting (OR 1.08; 95% CI 1.03 to 1.15) and thinness (OR 1.08; 95% CI 1.02 to 1.14), although the strength of these associations was weaker compared with children in urban areas. Opposite associations were found when considering the relationship between BFP participation and overweight/obesity among urban residents (BMIZ—OR 1.07; 95% CI 1.05 to 1.10) and rural residents (BMIZ—OR 0.91; 95% CI 0.87 to 0.95).

## Discussion

In this large longitudinal population-based study, we found that being enrolled in the Brazilian conditional cash transfer programme was associated with a decreased chance of stunting among under-5 children. This protective effect was particularly pronounced in children whose mothers had lower educational levels (≤3 years of formal education) and those living in rural areas. However, BFP participation was also linked to a higher likelihood of wasting and thinness, though this association weakened among children from households with lower maternal education and rural residence. Additionally, we observed a mixed association between BFP participation and overweight/obesity: an increased likelihood among children whose mothers had 8 or more years of education and lived in urban areas but a protective effect in those with less than 3 years of education and living in rural areas.

Our study is consistent with a previous Brazilian study that reported a 26% higher likelihood of adequate HAZ (or not stunted; length/height-for-age z-score≥−2) among under-5 children enrolled in the BFP compared with non-beneficiaries, which was conducted in 22 375 under-5 children, between 2005 and 2006.^17^ However, our study provides a larger sample size, which allowed us to assess the impact of BFP across various population subgroups.

Recent systematic reviews suggest uncertainty about the benefits of CCTs for improving child nutrition.^19 30^ Even within Brazil, the findings present a conflicting scenario.^22 23^ In a longitudinal study with Brazilian Amazonian children, participation in BFP was not associated with changes in continuous HAZ indicator and stunting over the follow-up period.^22^ A study involving Brazilian children and adolescents revealed that the BFP did not demonstrate any significant association with stunting.^23^ In contrast, data from the Pelotas Birth Cohort^43^ indicated a positive association between BFP in length-for-age z-score (LAZ) in 2 year-old children. In that study, the expected difference in LAZ was more pronounced among recipients of over R$1000 (approximately US$183.00) in benefits over 24 months (LAZ: −0.20 (95% CI −0.33 to –0.08)).^43^ In a different context, a study conducted in the Philippines showed higher odds of stunting (OR 1.43; 95% CI 1.08 to 1.91) among beneficiaries of the Pantawid Pamilyang Pilipino Program (4Ps) compared with non-4Ps children.^44^ In the same study, when comparing two specific areas, it was found that children participating in the 4Ps programme had a lower likelihood of stunting in an area with active parental engagement in feeding practice sessions (OR 0.41; 95% CI 0.23 to 0.71).^44^

We observed a more pronounced association between BFP and a reduced chance of stunting in children from low-education maternal levels and rural areas. This agrees with the findings of Ruel and colleagues,^45^ who indicate that urban residents rely on cash income for food and other basic needs, as they cannot turn to agriculture for short-term food or income and often lack access to land.

In this study, we also observed that participation in the BFP was associated with a higher likelihood of wasting. Similar to our findings, higher prevalence rates of wasting have been reported in children aged 24–59 months who received the BFP compared with non-beneficiaries.^7^ Conversely, another study found no statistically significant association between BFP participation and wasting among children under 5, despite observing a high prevalence ratio (PR 1.4; 95% CI 0.7 to 2.6).^46^

Stunting and wasting are distinct forms of undernutrition.^7^ Although both are rooted in poverty, they do not necessarily manifest similarly. Decreased weight-for-height or wasting is typically a short-term condition, often triggered by acute food shortages or infectious diseases, and can be reversed with adequate nutrition, healthcare access, vaccination, proper hygiene practices and access to clean water and sanitation.^29 32^ Weight-for-height changes are often less extreme, possibly due to stricter biological regulation.^32^ Both low height-for-age and low weight-for-height in childhood share common underlying factors, but long-term exposure to inadequate nutrition, living in persistently inadequate environmental conditions, with limited access to healthcare and education, and recurrent infections increase the risk of chronic malnutrition, manifested as stunting.^28 29 47^

We included several potential confounders in the relationship between BFP and malnutrition, including housing characteristics such as access to piped water and sewage systems, to ensure balance between BFP and non-BFP groups. Due to the unavailability of data, we were unable to explore potential sources of heterogeneity, such as access to healthy food, breastfeeding and complementary feeding, proximity to services, vaccination, supplementation and comorbidities. These factors could provide additional insight into the distinct relationships between BFP, stunting and wasting.

We also found that being enrolled in the BFP was associated with increased overweight/obesity among children whose mothers had higher education and lived in urban areas, but had a protective effect among those with less education and living in rural areas. A higher prevalence of overweight/obesity was also observed in children aged 24–59 months who were recipients of BFP compared with non-beneficiaries.^7^ Previous studies in Brazil have shown mixed results; some indicate a lower prevalence of overweight/obesity among beneficiaries compared with non-beneficiaries,^23^ while others found no statistically significant association.^46^ Similarly, a recent review found varying effects of CCTs on overweight and obesity across age groups, with some programmes showing no impact while others potentially reducing the risk in children, adolescents and adults.^30^ These results may arise from differences in study populations and methodologies. Our study contributes to this body of evidence by showing that the effects of BFP on childhood overweight/obesity are not homogeneous but rather influenced by socioeconomic and geographic factors.

The mixed findings in our study regarding stunting and overweight—with BFP participation particularly reducing stunting among children of less educated mothers in rural areas and increasing overweight among children of more educated mothers in urban areas—may be explained by the overlapping stages of the rapid nutrition transition in Brazil.^48^ The nutrition transition has evolved at different paces and times across regions, countries and even population subgroups.^49^ Previous studies have identified an advanced stage of nutrition transition in Brazil, characterised by an increasing double burden of malnutrition, where forms of undernutrition coexist with overweight and obesity at the population,^50^ household^51^ and individual levels,^7^ particularly among the most socioeconomically vulnerable.

Some studies have found a notably high prevalence of ultraprocessed food consumption among BFP beneficiaries aged 6–24 months, while continued breastfeeding for at least 2 years was linked to lower intake of these foods.^52^ Another study indicated that adult and adolescent beneficiaries of the BFP tend to consume higher levels of energy and macronutrients, such as carbohydrates, fats and added sugars.^25^ In contrast, other research found that BFP beneficiaries consumed fewer processed and ultraprocessed foods.^24^ These conflicting findings underscore the need for further research to better understand the relationship between conditional cash transfers and childhood overweight/obesity. CCTs programmes may have heterogeneous effects across different contexts and even different subpopulations.^15 16 33^ Although randomised controlled trials (RCTs) are considered the gold standard for causal inference, they are often not a feasible design for evaluating the effects of CCTs, such as the BFP, primarily due to the large-scale and non-randomised nature of their implementation. Moreover, RCTs tend to have smaller sample sizes, which may limit their ability to capture effects within subpopulations—a key advantage of large observational and associative studies for policy evaluation in population health.

By demonstrating the association between BFP and childhood overweight/obesity across socioeconomic and geographic contexts, our study underscores the need for targeted policy approaches. Specifically, integrating BFP with nutrition education, improving access to healthy foods and implementing regulatory measures on ultraprocessed food consumption could be particularly relevant for urban beneficiaries, where the risk appears to be greater.

Expanding access to and utilisation of primary healthcare services is crucial for reducing health and nutrition inequalities in childhood and is a key component of BFP through its health-related conditionalities.^3 28 53^ In Brazil, primary healthcare plays a vital role as the gateway to the Unified Health System, with a notable increase in municipal coverage through the Family Health Strategy over time.^28 54^

We hypothesised that the BFP affects child nutrition by enhancing food consumption and promoting compliance with programme conditionalities such as vaccination, child nutritional monitoring, promotion of breastfeeding and complementary feeding practices.^18 26 39^ Beyond the importance of availability and accessibility of healthy foods, the changing dietary patterns pose challenges as the low-nutritional-value food industry targets the ‘new consumer class’, hindering healthier consumption among BFP beneficiaries.^17^

Our findings align with previous research on the impact of CCTs on child health in LMICs, showing significant differences among subgroups based on socioeconomic status indicators.^15 16 33 42^ Drawing definitive conclusions regarding the association between CCTs and nutritional indicators in children remains challenging based on current evidence. This challenge arises from the heterogeneity of the studies, including variations in sample size, analytical methods, population demographics and age ranges.^19 30^ Certainly, the health and nutritional status of a population are undeniably interconnected with various determinants spanning multiple domains, including poverty, health inequalities and the health impacts of environmental inequalities.^11^ Therefore, interventions such as the BFP may promote the improvement of nutritional status, especially among the most vulnerable populations.

### Strengths and limitations

We comprehensively examined various socioeconomic variables at family and individual levels and a diverse range of risk factors rarely found in administrative data sets. To address observed confounding factors and ensure a more appropriate comparison group, we employed kernel-based PS weighting. This PS-based analytical method is widely used to address a common challenge in policy analysis, where treated and untreated (or control) groups often differ beyond their treatment status, making direct comparisons using traditional methods less reliable.^40^ Our study identified a well-balanced distribution of observed covariates between beneficiary and non-beneficiary groups after kernel weighting, suggesting that the observed associations are likely attributable to the BFP (online supplemental 1, eTable 3). Our large data set encompasses Brazil’s poorest population, which is eligible for social programmes, providing a unique opportunity to test specific hypotheses and enhance the robustness of our analyses. It allowed us to apply the same approach across different population subgroups, which was only possible due to the size of our data set. Additionally, it enabled us to explore the association between two different BFP receipt statuses and their impact on malnutrition. However, limitations should be acknowledged. Selection bias related to the BFP has been reported in prior studies, following similar approaches to balance beneficiary and non-beneficiary groups based on observable characteristics.^15 33^ We have employed subgroup analyses to mitigate this and strengthen the evidence. In our study, the BFP is treated as a binary variable, and we did not explore nuances related to the amount received and poverty levels. Other limitations may be related to errors in the collection and entry of anthropometric measurements in SISVAN. However, the quality of SISVAN anthropometric data for children under 5 years of age has improved over time.^35^

## Conclusion

The study highlights the positive impact of BFP participation on child nutritional outcomes, emphasising its potential to reduce stunting and lower rates of overweight and obesity, among the most vulnerable children. However, BFP participation was also associated with a higher risk of overweight/obesity among less vulnerable children. We speculate that these results reflect overlapping stages of Brazil’s rapid nutrition transition, which is associated with the rising levels of double burden of malnutrition (DBM). However, further investigations are needed to better understand the underlying mechanisms involved in this condition.

These insights provide valuable guidance for policymakers in developing effective strategies to combat child malnutrition. These strategies involve monitoring adherence to BFP conditionalities in terms of frequency, quality of care provided and identification of potential access barriers to education and health services. Additionally, a key challenge for BFP management is addressing obesity among beneficiaries, particularly in urban areas, which may require greater integration of the programme with policies and initiatives related to nutrition education, access to healthy foods and taxation of ultraprocessed foods.

## Supplementary material

10.1136/bmjgh-2024-018431 online supplemental file 1

## Data Availability

Data are available upon reasonable request.
