# Supplementary material for: Brazil’s Bolsa Família conditional cash transfer and child malnutrition: a nationwide birth cohort study
Source: BMJ Glob Health. 2025 Jul 20;10(7):e018431. doi: 10.1136/bmjgh-2024-018431 (PMC12278147; doi:10.1136/bmjgh-2024-018431)
Supplement: online supplemental file 1 [file bmjgh-10-7-s001.docx]

Additional information: **Brazil's Bolsa Família Conditional Cash Transfer and Child Malnutrition: A Nationwide Birth Cohort Study**

Ila R. Falcão et al.

**SUPPLEMENT 1**

1. THE CIDACS RECORD LINKAGE (CIDACS-RL)

CIDACS-RL ^1^ ^2^ is a linkage tool of individual records in two stages, using identifiers. In this case, the mother’s name, age or date of birth, and municipality of residence were used. The first stage was the deterministic linkage, and the second was based on a similarity index generated from these ^1^. All the linkage procedures were carried out at the Center for Data and Knowledge Integration for Health (CIDACS), Fiocruz ^3^, in a strict data protection environment, and compliance with ethical and legal standards ^4^.

Two linkage approaches were considered: deterministic linkage (or merge) when the Social Identification Number (NIS) was populated (Approach 1) in both databases and non-deterministic linkage (Approach 2) for records with missing NIS. A total of 257,049,913 records were linked, representing 83.66% of the data (the sum of linked records from both Approaches 1 and 2, including all follow-up records from SISVAN).

The results for the accuracy of the linkage, based on the optimal cut-off point (0.941), were as follows: specificity 93.80% and sensitivity 97.20%. The receiver operating characteristic (ROC) curve can be seen in eFigure 1 below.


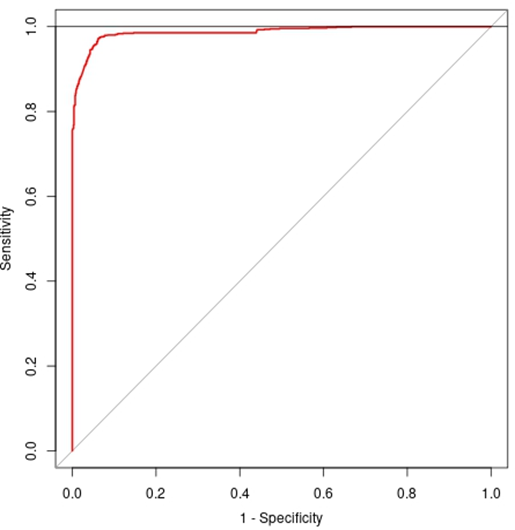


**eFigure 1.** ROC curve of linkage between the 100 million cohort (POP100 V2) and SISVAN-Antro (Approach 2).

Source: Prepared by the CIDACS Data Production Center.

It is important to note that linkage was performed on the complete dataset, which includes children, adults, and elderly individuals, resulting in 43,058,103 linked records for children (eFigure2).

2. DETAILED INFORMATION ABOUT ELIGIBLE STUDY POPULATION


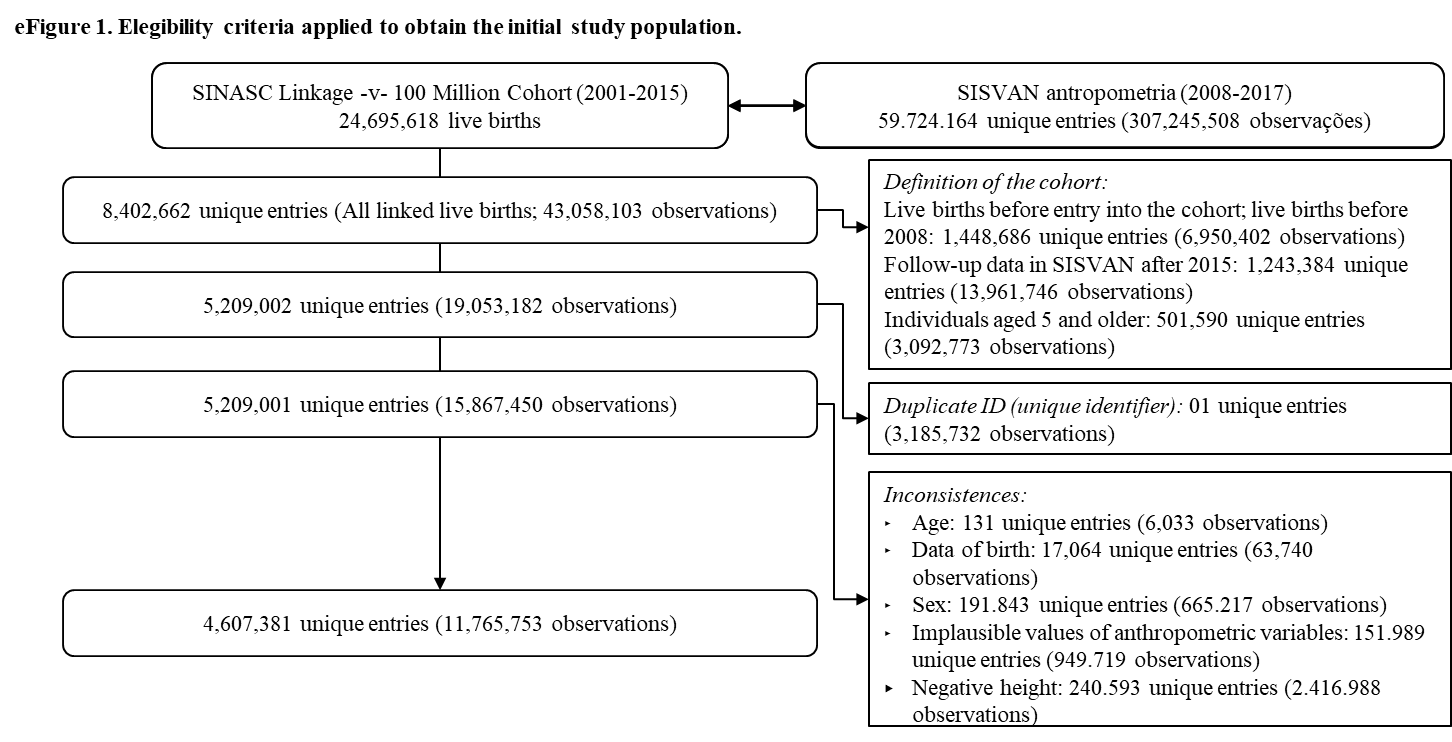


**eFigure 2.** Elegibility criteria applied to obtain the initial study population.

3. THE BOLSA FAMILIA PROGRAM (BFP)

The BFP determines eligibility based on the per capita income of families registered in the Cadastro Único for Social Programs of the Brazilian Federal Government (*CadÚnico)* and their composition ^5^. Families classified as extremely poor are eligible regardless of composition and those with low income qualify if they include at least one individual from priority groups, such as children ^6^. Ideally, cash disbursements are directed to women, contingent on compliance with specific program requirements (conditionalities) such as the utilization of healthcare services throughout childhood, pregnancy and in the postpartum period ^7^ ^6^.

4. PROPENSITY SCORE

The propensity score (PS) was characterized as the probability of being a Bolsa Familia (BFP) beneficiary (or not), conditioned on the baseline characteristics observed (potential confounders) ^8^. Our analysis involved a PS estimate through a logistic model, to estimate the probability of receiving BFP based on maternal variables (eTable 1).

| **eTable 1.** Variables used in this study, Brazil, 2008-2015. | | |
| --- | --- | --- |
| **Variable** | **Classification** | **Database** |
| **Variables used to calculate the propensity score** |  |  |
| *Sociodemographic characteristics* |  |  |
| Region of residence | 5 categories related to the Brazilian regions (North, Northeast, South, Southeast and Central-West) | 100 million cohort (Cadastro Único data) |
| Self-reported race | Asian descent, Black, Parda, Indigenous and White |  |
| Level of education | ≥8 years of education; 4-7 years; ≤3 years | SINASC |
| Marital status | Partner; no partner |  |
| *Housing characteristics* |  |  |
| Construction materials | Brick, wood, or other | 100 million cohort (Cadastro Único data) |
| Water supply | Public network, well, or other |  |
| Electricity | Housing with an electricity meter; Housing without a meter |  |
| Waste collection | Collected, burned, buried, or other |  |
| Sanitary system | Public network, septic tank, or other |  |
| Overcrowding | No (≤2 inhabitants/room); Yes (>2 inhabitants/room) |  |
| *Year of entry* | 2008/2011; 2012/2015 |  |
| **Variables used in the adjusted models** |  |  |
| Low Birth Weight | Yes: <2500g; No: ≥2500g | SINASC |
| Sex assigned at birth | Category: Male or female |  |
| Mother’s age on delivery | Categorized: <20 years old; 20-34 years old; ≥35 years old |  |
| Type of delivery | Vaginal or C-section |  |

The common support graph and descriptive analysis of the propensity score can be found in eFigure 2 and eTable 2. These analyses refer to the propensity score calculated for Brazil, considering a population of 3,116,138 under-5 children born at term.

The differences between beneficiaries and non-beneficiaries according to the PS variables were evaluated before and after kernel weighting (eTable 3). We observed that these differences were reduced after weighting.

.**
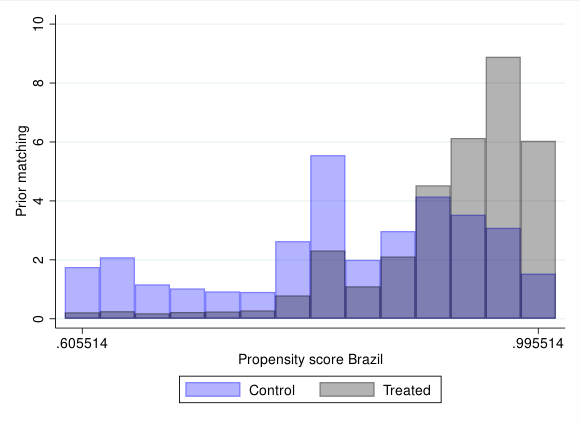
**

**eFigure 3.** Common support graph of the propensity score estimated for Brazil, considering a population of 3,116,138 under-5 children born at term.

| **eTable 2.** Propensity score description in accordance with the confounding covariates observed, Brazil, 2008 to 2015. | | |
| --- | --- | --- |
| **Propensity score** | **Brazil** | |
|  | **Non-BFP (N=270,753)** | **BFP (N=2,845,385)** |
| Average | 0.831 | 0.921 |
| Standard deviation | 0.111 | 0.074 |
| Minimum | 0.591 | 0.591 |
| 25^th^ Percentile | 0.787 | 0.898 |
| Median | 0.842 | 0.944 |
| 75^th^ Percentile | 0.920 | 0.974 |
| Maximum | 0.998 | 0.998 |

| **eTable 3.** Description of the variables with complete data used for the propensity score, according to receipt of Bolsa Família Program (BFP), for all live births between 2008-2015 of the women who entered the cohort in Brazil. | | | | | | |
| --- | --- | --- | --- | --- | --- | --- |
| **Propensity score variables used** | **Relative frequency and non-weighted proportion (1)** | | | **Relative frequency and weighted proportion (2)** | | |
|  | **Non-BFP (N= 270,753)** | **BFP (N=2,845,385)** | **Diff**  **(3)** | **Non-BFP (N=270,728)** | **BFP (N=2,841,210)** | **Diff**  **(3)** |
|  | **%** | **%** |  | **%** | **%** |  |
| ***Sociodemographic characteristics*** |  |  |  |  |  |  |
| **Level of education** |  |  |  |  |  |  |
| >=8 years of education | 68.24 | 50.3 | -18.0 | 50.8 | 50.3 | -0.5 |
| 4-7 years | 27.0 | 37.8 | 10.8 | 38.1 | 37.8 | -0.3 |
| <=3 years | 4.8 | 11.9 | 7.2 | 11.1 | 11.9 | 0.8 |
| **Race/skin color (self-reported)** |  |  |  |  |  |  |
| Asian descent | 0.4 | 0.4 | 0.0 | 0.4 | 0.4 | 0.0 |
| Black | 3.0 | 3.8 | 0.8 | 3.8 | 3.8 | -0.1 |
| Parda | 45.8 | 64.4 | 18.5 | 64.4 | 64.4 | 0.0 |
| Indigenous | 0.2 | 0.7 | 0.5 | 0.5 | 0.6 | 0.1 |
| White | 50.6 | 30.8 | -19.8 | 30.8 | 30.8 | 0.0 |
| **Marital status** |  |  |  |  |  |  |
| Partner | 45.0 | 38.9 | -6.1 | 37.9 | 38.9 | 1.0 |
| No partner | 55.0 | 61.1 | 6.1 | 62.1 | 61.1 | -1.0 |
| **Region of residency** |  |  |  |  |  |  |
| North | 5.7 | 12.7 | 7.0 | 12.2 | 12.6 | 0.4 |
| Northeast | 19.3 | 41.9 | 22.7 | 43.0 | 42.0 | -1.0 |
| Southeast | 35.5 | 28.6 | -6.8 | 28.3 | 28.7 | 0.4 |
| South | 31.0 | 10.4 | -20.6 | 10.3 | 10.4 | 0.1 |
| Central-West | 8.6 | 6.4 | -2.2 | 6.2 | 6.4 | 0.2 |
| ***Housing characteristics*** |  |  |  |  |  |  |
| **Construction materials** |  |  |  |  |  | 0.0 |
| Brick | 83.2 | 78.0 | -5.2 | 78.3 | 78.0 | -0.2 |
| Wood, or other | 16.8 | 22.0 | 5.2 | 21.7 | 22.0 | 0.2 |
| **Water supply** |  |  |  |  |  |  |
| Public network | 83.1 | 70.9 | -12.3 | 70.9 | 70.9 | 0.1 |
| Well, or other | 16.9 | 29.1 | 12.3 | 29.1 | 29.1 | -0.1 |
| **Electricity** |  |  |  |  |  |  |
| Home with electricity meter | 93.7 | 85.3 | -8.4 | 84.9 | 85.4 | 0.5 |
| Home without a meter | 6.3 | 14.7 | 8.4 | 15.1 | 14.6 | -0.5 |
| **Waste collection** |  |  |  |  |  |  |
| Collected | 89.0 | 75.9 | -13.2 | 76.1 | 75.9 | -0.2 |
| Burned, buried, or other | 11.0 | 24.1 | 13.2 | 23.9 | 24.1 | 0.2 |
| **Sanitation system** |  |  |  |  |  |  |
| Public network | 54.2 | 40.4 | -13.7 | 40.4 | 40.4 | 0.0 |
| Septic tank, or other | 45.8 | 59.6 | 13.7 | 59.6 | 59.6 | 0.0 |
| **Overcrowding** |  |  |  |  |  |  |
| No (≤2 inhabitants/room) | 97.7 | 92.2 | -5.6 | 93.0 | 92.2 | -0.8 |
| Yes (>2 inhabitants/room) | 2.3 | 7.8 | 5.6 | 7.0 | 7.8 | 0.8 |
| ***Year of entry into the cohort baseline*** |  |  |  |  |  | 0.0 |
| 2008/2011 | 21.7 | 50.7 | 28.9 | 50.8 | 50.7 | -0.1 |
| 2012/2015 | 78.3 | 49.3 | -28.9 | 49.2 | 49.3 | 0.1 |
| (1) Before kernel weighting | | | | | | |
| (2) After kernel weighting | | | | | | |
| (3) Difference in proportion of each category between Bolsa Familia beneficiaries and non-beneficiaries. | | | | | | |

5. MISSING DATA

A descriptive analysis of missing data, based on the population of 3,769,490 before excluding incomplete records, is provided in eTable 4 and 5.

| **eTable 4.** Missing data for variables used in this study before exclusion, by Bolsa Família (BFP) status, Brazil, 2008 to 2015. | | | |
| --- | --- | --- | --- |
|  | **Missing data** | | |
|  | **Brazil (N=3,769,490)**  **N (%)** | **Non-BFP (N=309,497)**  **N (%)** | **BFP**  **(N=3,459,993)**  **N (%)** |
| *Propensity score variables* |  |  |  |
| Region of residence | 0 (0.0) | 0 (0.0) | 0 (0.0) |
| Level of education | 66,451 (1.8) | 4,621 (1.5) | 61,830 (1.8) |
| Race | 98 (0.0) | 39 (0.0) | 59 (0.0) |
| Marital status | 53,607 (1.4) | 3,969 (1.3) | 49,638 (1.4) |
| Area of residency | 81 (0.0) | 11 (0.0) | 70 (0.0) |
| Construction materials | 116,391 (3.1) | 11,810 (3.8) | 104,581 (3.0) |
| Water supply | 116,410 (3.1) | 11,815 (3.8) | 104,595 (3.0) |
| Electricity | 116,378 (3.1) | 11,815 (3.8) | 104,563 (3.0) |
| Waste collection | 116,420 (3.1) | 11,815 (3.8) | 104,605 (3.0) |
| Sanitation system | 356,220 (9.5) | 21,075 (6.8) | 335,145 (9.7) |
| Overcrowding | 321,993 (8.5) | 22,450 (7.3) | 299,543 (8.7) |
| Year of entry | 0 (0.0) | 0 (0.0) | 0 (0.0) |
| *Variables used in the adjusted models* |  |  |  |
| Low Birth Weight | 252,772 (6.71) | 16,304 (5.3) | 236,468 (6.8) |
| Sex assigned at birth | 0 (0.0) | 0 (0.0) | 0 (0.0) |
| Mother’s age on delivery | 58 (0.0) | 7 (0.0) | 51 (0.0) |
| Type of delivery | 4,166 (0.1) | 276 (0.1) | 3,890 (0.1) |

A descriptive analysis in accordance with the missing pattern is provided in eTable 2.

| **eTable 5.** **Distribution of missing data, in accordance with the patterns (1), Brazil, 2004 to 2015.** | |
| --- | --- |
| **Missing patterns** | **N (%)** |
| Completeness | 3,116,138 (82.7) |
| Assorted pattern | 653,352 (17.3) |
| Monotonicity | 0 (0.0) |
| Total | 3,769,490 (100.0) |
| (1) Completeness: all of the variables with information; Assorted pattern: a minimum of one variable with missing data; Monotonicity: all of the incomplete variables. | |

To evaluate the influence of missing data on the analyses, we included missing data as a separate category for each variable in the propensity score estimation. We then followed the same steps as in the primary analysis: kernel matching and weighted logistic and multinomial regressions were conducted for each outcome. The results are shown in eTable 6.

| **eTable 6.** Adjusted and Weighted coefficients of Bolsa Familia beneficiaries on nutritional indicators of children born in Brazil between 2008 and 2015, considering propensity score variables with missing data category. | | | |
| --- | --- | --- | --- |
| **Outcome/Estimate** | **OR (95% CI)** | **Robust Standard Error** | **p-value** |
| **Adjusted and weighted models (N = 3,503,907)** |  |  |  |
| *Model 1 (1): Categorized Z-score for length/height-for-age (LAZ/HAZ)* |  |  |  |
| Stunting (LAZ/HAZ < -2) | 0.83 (0.81; 0.85) | 0.009 | <0.001 |
| *Model 2 (2): Categorized Z-score for weight-for-age* |  |  |  |
| Underweight (WAZ< -2) | 0.90 (0.87; 0.93) | 0.015 | <0.001 |
| High weight (WAZ>2) | 1.12 (1.10; 1.15) | 0.013 | <0.001 |
| *Model 3 (2): Categorized Z-score for weight-for-length/height (WHZ)* |  |  |  |
| Wasting (WHZ< -2) | 1.19 (1.16; 1.22) | 0.016 | <0.001 |
| Overweight risk (1<WHZ≤2) | 0.94 (0.93; 0.95) | 0.007 | <0.001 |
| Overweight/Obesity (WHZ>2) | 0.97 (0.95; 0.99) | 0.009 | <0.01 |
| *Model 4 (2): Categorized Z-score for body mass index for age (BMIZ)* |  |  |  |
| Thinness (BMIZ< -2) | 1.18 (1.15; 1.21) | 0.015 | <0.001 |
| Overweight rink (1<BMIZ≤2) | 0.97 (0.96; 0.99) | 0.007 | <0.001 |
| Overweight/Obesity (BMIZ>2) | 1.03 (1.01; 1.05) | 0.009 | <0.01 |
| (1) Logistic regression results: analysis weighted and adjusted for gestational age, sex assigned at birth, mother's age at birth, and type of delivery. | | | |
| (2) Multinomial regression results: analysis weighted and adjusted for gestational age, sex assigned at birth, mother's age at birth, and type of delivery. | | | |

6. CRUDE MODELS, ADJUSTED MODELS, AND ADJUSTED AND WEIGHTED MODELS

The results of the crude analyses are presented below (eTable 7). We also present the adjusted but unweighted analyses. A more detailed description of the adjusted and weighted analysis results shown in Figure 2 of the article is provided in the same table.

| **eTable 7.** Coefficients of crude, adjusted and adjusted and weighted binary and multinomial regressions of Bolsa Familia beneficiaries on nutritional indicators of children born in Brazil between 2008 and 2015. | | | |  |
| --- | --- | --- | --- | --- |
| **Crude models (N =3,116,138)** | **OR (95% CI)** | **Robust Standard Error** | **p-value** |  |
| Model 1: Categorized Z-score for length/height-for-age (LAZ/HAZ) |  |  |  |  |
| Stunting (LAZ/HAZ < -2) | 1.01 (1.00; 1.03) | 0.008 | 0.147 |  |
| Model 2: Categorized Z-score for weight-for-length/height (WHZ) |  |  |  |  |
| Wasting (WHZ< -2) | 1.62 (1.59; 1.65) | 0.016 | <0.001 |  |
| Overweight risk (1<WHZ≤2) | 0.88 (0.87; 0.89) | 0.004 | <0.001 |  |
| Overweight/Obesity (WHZ>2) | 0.98 (0.97; 1.00) | 0.006 | 0.012 |  |
| Model 3: Categorized Z-score for body- mass-index-for-age (BMIZ) |  |  |  |  |
| Thinness (BMIZ< -2) | 1.58 (1.55; 1.61) | 0.015 | <0.001 |  |
| Overweight risk (1<BMIZ≤2) | 0.93 (0.92; 0.94) | 0.005 | <0.001 |  |
| Overweight/Obesity (BMIZ>2) | 1.07 (1.06; 1.08) | 0.007 | <0.001 |  |
| **Adjusted and weighted models (N = 3,106,614)** |  |  |  |  |
| Model 1 (1): Categorized Z-score for length/height-for-age (LAZ/HAZ) |  |  |  |  |
| Stunting (LAZ/HAZ < -2) | 0.83 (0.81; 0.85) | 0.009 | <0.001 |  |
| Model 2 (2): Categorized Z-score for weight-for-length/height (WHZ) |  |  |  |  |
| Wasting (WHZ< -2) | 1.19 (1.16; 1.23) | 0.017 | <0.001 |  |
| Overweight risk (1<WHZ≤2) | 0.95 (0.93; 0.96) | 0.007 | <0.001 |  |
| Overweight/Obesity (WHZ>2) | 0.97 (0.95; 0.99) | 0.009 | 0.004 |  |
| Model 3 (2): Categorized Z-score for body mass index for age (BMIZ) |  |  |  |  |
| Thinness (BMIZ< -2) | 1.20 (1.17; 1.23) | 0.016 | <0.001 |  |
| Overweight rink (1<BMIZ≤2) | 0.98 (0.97; 1.00) | 0.008 | 0.016 |  |
| Overweight/Obesity (BMIZ>2) | 1.03 (1.01; 1.05) | 0.010 | <0.01 |  |
| **Adjusted but not weighted models (3) (N = 3,110,772)** |  |  |  |  |
| Model 1 (3): Categorized Z-score for length/height-for-age (LAZ/HAZ) |  |  |  |  |
| Stunting (LAZ/HAZ < -2) | 0.91 (0.90; 0.92) | 0.007 | <0.001 |  |
| Model 2 (4): Categorized Z-score for weight-for-length/height (WHZ) |  |  |  |  |
| Wasting (WHZ< -2) | 1.30 (1.27; 1.32) | 0.013 | <0.001 |  |
| Overweight risk (1<WHZ≤2) | 1.02 (1.01; 1.03) | 0.006 | <0.01 |  |
| Overweight/Obesity (WHZ>2) | 1.14 (1.12; 1.15) | 0.008 | <0.001 |  |
| Model 3 (4): Categorized Z-score for body mass index for age (BMIZ) |  |  |  |  |
| Thinness (BMIZ< -2) | 1.32 (1.30; 1.35) | 0.013 | <0.001 |  |
| Overweight rink (1<BMIZ≤2) | 1.07 (1.06; 1.08) | 0.006 | <0.001 |  |
| Overweight/Obesity (BMIZ>2) | 1.24 (1.23; 1.26) | 0.008 | <0.001 |  |
| (1) Logistic regression results: analysis adjusted for all propensity score variables and sex assigned at birth, mother's age at birth, low birthweight, and type of delivery. | | | |  |
| (2) Multinomial regression results: analysis adjusted for all propensity score variables and sex assigned at birth, mother's age at birth, low birthweight, and type of delivery. | | | |  |
| (3) Logistic regression results: analysis weighted and adjusted for sex assigned at birth, mother's age at birth, low birthweight, and type of delivery. | | | |  |
| (4) Multinomial regression results: analysis weighted and adjusted for sex assigned at birth, mother's age at birth, low birthweight, and type of delivery. | | | |  |

7. SUBGROUP ANALYSIS: ALTERNATIVE APPROACH

We identified groups that changed their beneficiary status between two follow-ups. We selected the first and last follow-ups for each child from birth to 59 months (eFigure 3).

**eFigure 4**. Eligibility criteria applied to obtain the study population with two or more observations for children under 5 years of age.


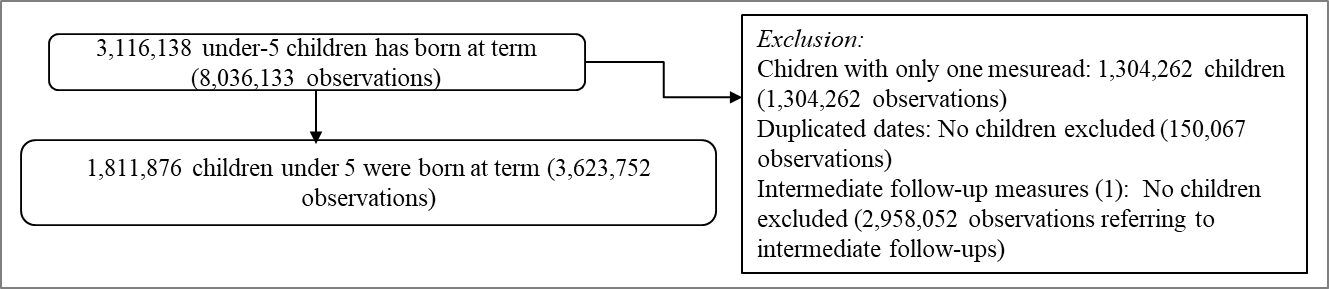


(1) Two follow-up visits were considered per child: the first and the last follow-up for each child.

The groups were defined based on benefit receipt as follows: i. Never received – children who did not receive the benefit at either the first or the last follow-up; ii. Started receiving – children who did not receive the benefit at the first follow-up but began receiving it after and continued until the last follow-up; iii. Always received – children who received the benefit at both measures.

After defining the two BFP exposure groups, we restricted the dataset to the child’s last follow-up and assessed the association between BFP receipt and nutritional outcomes, following the same steps as in the primary analysis (eTable 8). The idea of assessing the association of BFP with nutritional indicators by subgroups, while also considering variations in receipt, was to select exposure groups that are more comparable to the non-exposure groups, given that we do not have family income data to analyze subgroups by poverty and extreme poverty.

| **eTable 8.** Adjusted and weighted coefficients of Bolsa Família (BFP) beneficiaries on nutritional indicators of children born in Brazil between 2008 and 2015, considering variations in BFP receipt. | | |
| --- | --- | --- |
| **Outcome/Exposure** | **Always received vs. never received BFP** | **Started receiving after the first follow-up vs. never received BFP** |
| **Adjusted and weighted models (N = 1,808,612)** | **OR (95% CI)** | **OR (95% CI)** |
|  |  |  |
| *Model 1 (1): Categorized Z-score for length/height-for-age (LAZ/HAZ)* |  |  |
| Stunting (LAZ/HAZ < -2) | 1.07***  (1.04; 1.10) | 0.83***  (0.80; 0.87) |
| *Model 2 (2): Categorized Z-score for weight-for-age* |  |  |
| Underweight (WAZ< -2) | 1.10***  (1.06; 1.15) | 0.94  (0.88; 1.00) |
| High weight (WAZ>2) | 1.07***  (1.05; 1.10) | 1.08***  (1.05; 1.12) |
| *Model 3 (2): Categorized Z-score for weight-for-length/height (WHZ)* |  |  |
| Wasting (WHZ< -2) | 1.49***  (1.44; 1.53) | 1.32***  (1.27; 1.38) |
| Overweight risk (1<WHZ≤2) | 0.96***  (0.94; 0.97) | 0.91***  (0.89; 0.93) |
| Overweight/Obesity (WHZ>2) | 1.09***  (1.06; 1.11) | 1.00  (0.97; 1.03) |
| *Model 4 (2): Categorized Z-score for body mass index for age (BMIZ)* |  |  |
| Thinness (BMIZ< -2) | 1.43***  (1.39; 1.48) | 1.29***  (1.24; 1.34) |
| Overweight rink (1<BMIZ≤2) | 1.00  (0.98; 1.01) | 0.94***  (0.92; 0.96) |
| Overweight/Obesity (BMIZ>2) | 1.14***  (1.12; 1.17) | 1.03*  (1.00; 1.07) |
| (1) Logistic regression results: analysis weighted and adjusted for gestational age, sex assigned at birth, mother's age at birth, and type of delivery. | | |
| (2) Multinomial regression results: analysis weighted and adjusted for gestational age, sex assigned at birth, mother's age at birth, and type of delivery. | | |
| *p-value <0.05; ** p-value <0.01; ***p-value <0.001 | | |

8. SUBGROUP ANALISIS: PRIMIPAROUS WOMEN

The analytical steps (propensity score estimation, kernel matching and weighted logistic regression) were conducted separately within each area of residence (urban or rural) and level of education. In our study, we did not include the area of residence variable in the PS estimation for the subgroup analyses based on area, and those based on level of education.

Furthermore, we selected only women with no history of previous live births to examine the association of BFP with nutritional outcomes among primiparous women, using weighted and adjusted models (eTable 9). This approach was chosen because there are two income thresholds for Bolsa Família eligibility: the extreme poverty threshold, which makes a family eligible regardless of family composition, and the poverty threshold, which depends on family composition (children under 5 and school-attending children and adolescents) for program eligibility.

| **eTable 9.** Adjusted and Weighted coefficients of Bolsa Familia beneficiaries on nutritional indicators of children born in Brazil between 2008 and 2015, by subgroup of primiparous women. | | | |
| --- | --- | --- | --- |
| **Outcome/Estimate** | **OR (95% CI)** | **Robust Standard Error** | **p-value** |
| **Adjusted and weighted models (N=841,682)** |  |  |  |
| *Model 1 (1): Categorized Z-score for length/height-for-age (LAZ/HAZ)* |  |  |  |
| Stunting (LAZ/HAZ < -2) | 0.79 (0.76; 0.82) | 0.013 | <0.001 |
| *Model 3 (2): Categorized Z-score for weight-for-length/height (WHZ)* |  |  |  |
| Wasting (WHZ< -2) | 1.20 (1.16; 1.25) | 0.024 | <0.001 |
| Overweight risk (1<WHZ≤2) | 0.98 (0.96; 1.01) | 0.010 | 0.14 |
| Overweight/Obesity (WHZ>2) | 1.07 (1.04; 1.10) | 0.014 | <0.001 |
| *Model 4 (2): Categorized Z-score for body mass index for age (BMIZ)* |  |  |  |
| Thinness (BMIZ< -2) | 1.19 (1.15; 1.24) | 0.022 | <0.001 |
| Overweight rink (1<BMIZ≤2) | 1.02 (1.00; 1.04) | 0.011 | 0.09 |
| Overweight/Obesity (BMIZ>2) | 1.13 (1.10; 1.16) | 0.014 | <0.001 |
| (1) Logistic regression results: analysis weighted and adjusted for gestational age, sex assigned at birth, mother's age at birth, and type of delivery. | | | |
| (2) Multinomial regression results: analysis weighted and adjusted for gestational age, sex assigned at birth, mother's age at birth, and type of delivery. | | | |

9. ANALYSIS CONSIDERING CONTINUOS OUTCOMES

We assessed all indicators as continuous variables. The analytical steps (propensity score estimation, kernel matching and weighted linear regression) were conducted. The findings were in line with the main analysis presented in the article, taking into account the categorized variables.

| **eTable 10.** Coefficients of crude and adjusted kernel-weighted linear regressions of Bolsa Familia Beneficiaries on nutritional indicators of children born in Brazil between 2008 and 2015. | | | | | |
| --- | --- | --- | --- | --- | --- |
| **Crude models (1)** | β **(95% CI)** | **Robust Standard Error** | **p-value** | | **N** |
| Model 1: Z-score for length/height-for-age (LAZ/HAZ) | 0.04 (0.03; 0.05) | 0.003 | <0.001 | 3,116,138 | |
| Model 2: Z-score for weight-for-length/height (WHZ) | -0.15 (-0.16; -0.15) | 0.003 | <0.001 | 3,116,138 | |
| Model 3: Z-score for body mass index for age (BMIZ) | -0.11 (-0.12; -0.10) | 0.003 | <0.001 | 3,116,138 | |
| **Adjusted and weighted models (1)** |  |  |  |  | |
| Model 1: Z-score for length/height-for-age (LAZ/HAZ) | 0.08 (0.07; 0.09) | 0.005 | <0.001 | 3,104,447 | |
| Model 2: Z-score for weight-for-length/height (WHZ) | -0.07 (-0.08; -0.06) | 0.005 | <0.001 | 3,104,447 | |
| Model 3: Z-score for body mass index for age (BMIZ) | -0.04 (-0.05; -0.04) | 0.005 | <0.001 | 3,104,447 | |
| (1) Linear regression results: analysis weighted and adjusted for gestational age, sex of the live birth, mother's age at birth, and type of delivery. | | | | | |

10. REFERENCES

1. Barbosa GCG, Ali MS, Araujo B, et al. CIDACS-RL: a novel indexing search and scoring-based record linkage system for huge datasets with high accuracy and scalability. *BMC Medical Informatics and Decision Making* 2020; **20**(1): 289.

2. Almeida D, Gorender D, Ichihara MY, et al. Examining the quality of record linkage process using nationwide Brazilian administrative databases to build a large birth cohort. *BMC Medical Informatics and Decision Making* 2020; **20**(1): 173.

3. Barreto ML, Ichihara MY, Almeida BA, et al. The Centre for Data and Knowledge Integration for Health (CIDACS): Linking Health and Social Data in Brazil. *International journal of population data science* 2019; **4**(2): 1140.

4. Harron K, Dibben C, Boyd J, et al. Challenges in administrative data linkage for research. *Big Data & Society* 2017; **4**(2): 2053951717745678.

5. Gazola Hellmann A. How does Bolsa Familia work?: best practices in the implementation of conditional cash transfer programs in Latin America and the Caribbean: Inter-American Development Bank, 2015.

6. FGV. Bolsa Família: o que é e como funciona [Bolsa Família: What is it and how does it work]. 2018. <https://cps.fgv.br/bolsa-familia-o-que-e-e-como-funciona>.

7. Campello T, Neri MCO. Programa Bolsa Família uma década de inclusão e cidadania. Brasília: Ipea; 2013.

8. Rosenbaum PR, Rubin DB. Reducing Bias in Observational Studies Using Subclassification on the Propensity Score. *Journal of the American Statistical Association* 1984; **79**(387): 516-24.

**SUPPLEMENT 2**

**The RECORD statement – checklist of items, extended from the STROBE statement, that should be reported in observational studies using routinely collected health data.**

|  | **Item No.** | **STROBE items** | **Location in manuscript where items are reported** | **RECORD items** | **Location in manuscript where items are reported** |
| --- | --- | --- | --- | --- | --- |
| **Title and abstract** | | | | | |
|  | 1 | (a) Indicate the study’s design with a commonly used term in the title or the abstract (b) Provide in the abstract an informative and balanced summary of what was done and what was found | Abstract | RECORD 1.1: The type of data used should be specified in the title or abstract. When possible, the name of the databases used should be included.  RECORD 1.2: If applicable, the geographic region and timeframe within which the study took place should be reported in the title or abstract.  RECORD 1.3: If linkage between databases was conducted for the study, this should be clearly stated in the title or abstract. | Abstract |
| **Introduction** | | | | | |
| Background rationale | 2 | Explain the scientific background and rationale for the investigation being reported | Page 7 (lines 151-156) |  | Page 7 (lines 151-156) |
| Objectives | 3 | State specific objectives, including any prespecified hypotheses | Page 7 (lines 158-160) |  | Page 7 (lines 158-160) |
| **Methods** | | | | | |
| Study Design | 4 | Present key elements of study design early in the paper | Methods section: Study design and data sources (line 165-167) |  | Methods section: Study design and data sources (line 165-167) |
| Setting | 5 | Describe the setting, locations, and relevant dates, including periods of recruitment, exposure, follow-up, and data collection | Methods section: Study population (pages 8-9), Exposure (page 9), Study design and data sources (lines 176-178) |  | Methods section: Study population (pages 8-9), Exposure (page 9), Study design and data sources (lines 176-178) |
| Participants | 6 | *(a) Cohort study* - Give the eligibility criteria, and the sources and methods of selection of participants. Describe methods of follow-up  *Case-control study* - Give the eligibility criteria, and the sources and methods of case ascertainment and control selection. Give the rationale for the choice of cases and controls  *Cross-sectional study* - Give the eligibility criteria, and the sources and methods of selection of participants  *(b) Cohort study* - For matched studies, give matching criteria and number of exposed and unexposed  *Case-control study* - For matched studies, give matching criteria and the number of controls per case | Methods section: Exposure (page 9, eligibility criteria), Study population (page 8, methods of selection of participants), Study design and data sources (page 8, sources and methods of selection of participants) | RECORD 6.1: The methods of study population selection (such as codes or algorithms used to identify subjects) should be listed in detail. If this is not possible, an explanation should be provided.  RECORD 6.2: Any validation studies of the codes or algorithms used to select the population should be referenced. If validation was conducted for this study and not published elsewhere, detailed methods and results should be provided.  RECORD 6.3: If the study involved linkage of databases, consider use of a flow diagram or other graphical display to demonstrate the data linkage process, including the number of individuals with linked data at each stage. | 6.1: Methods section: Study population (page 8), Data linkage (Supplement 1, item 2)  6.2: Study design and data sources (page 8)  (CIDACS-RL tool, reference list - 32)  6.3: Study design and data sources (page 8);  Figure 1 (Results section); eFigure 2 (Supplement 1) |
| Variables | 7 | Clearly define all outcomes, exposures, predictors, potential confounders, and effect modifiers. Give diagnostic criteria, if applicable. | Methods section: page 9 (exposure), page 9 (outcomes), page 10 (lines 215-218 and 229-232, predictors, potential confounders, and effect modifiers). | RECORD 7.1: A complete list of codes and algorithms used to classify exposures, outcomes, confounders, and effect modifiers should be provided. If these cannot be reported, an explanation should be provided. | Methods section: page 9 (exposure), page 9 (outcomes), page 10 (lines 215-218 and 229-232, predictors, potential confounders, and effect modifiers). |
| Data sources/ measurement | 8 | For each variable of interest, give sources of data and details of methods of assessment (measurement).  Describe comparability of assessment methods if there is more than one group | Supplement 1 (eTable 1), and pre-specified protocol for this evaluation (citation order in manuscript - 34). |  | Supplement 1 (eTable 1), and pre-specified protocol for this evaluation (citation order in manuscript - 34). |
| Bias | 9 | Describe any efforts to address potential sources of bias | Study population (page 8), Study design and data sources (page 8), Statistical analysis (pages 10-11) |  | Study population (page 8), Study design and data sources (page 8), Statistical analysis (pages 10-11) |
| Study size | 10 | Explain how the study size was arrived at | Figure 1 (Results section), eFigure 2 (Supplement 1) |  | Figure 1 (Results section), eFigure 2 (Supplement 1) |
| Quantitative variables | 11 | Explain how quantitative variables were handled in the analyses. If applicable, describe which groupings were chosen, and why | Supplement 1 (eTable 1), |  | Supplement 1 (eTable 1) |
| Statistical methods | 12 | (a) Describe all statistical methods, including those used to control for confounding  (b) Describe any methods used to examine subgroups and interactions  (c) Explain how missing data were addressed  (d) *Cohort study* - If applicable, explain how loss to follow-up was addressed  *Case-control study* - If applicable, explain how matching of cases and controls was addressed  *Cross-sectional study* - If applicable, describe analytical methods taking account of sampling strategy  (e) Describe any sensitivity analyses | (a) Methods section: Statistical analysis (pages 10-11);  (b) Methods section: Statistical analysis (page 11);  (c) Methods section: Statistical analysis (lines 220-224);  (d) Not applicable (e) Supplement 1: eTables 8 and 9) |  | (a) Methods section: Statistical analysis (pages 10-11);  (b) Methods section: Statistical analysis (page 11);  (c) Methods section: Statistical analysis (lines 220-224);  (d) Not applicable (e) Supplement 1: eTables 8 and 9) |
| Data access and cleaning methods |  | .. | Methods section: Study population (lines 184-191), Study design and data sources (page 8), Statistical analysis (pages 7 and 8);  Supplement 1 (subsection 1; eTable 1);  Results section: Figure 1; Supplement 1: (eFigure 2) | RECORD 12.1: Authors should describe the extent to which the investigators had access to the database population used to create the study population.  RECORD 12.2: Authors should provide information on the data cleaning methods used in the study. | RECORD 12.1: Methods section: Study design and data sources (page 8); Supplement 1 (subsection 1; eTable 1);  RECORD 12.2:  Methods section: Study population (lines 184-191);  Results section: Figure 1; Supplement 1: (eFigure 2) |
| Linkage |  | .. | Supplement 1 (subsection 1) | RECORD 12.3: State whether the study included person-level, institutional-level, or other data linkage across two or more databases. The methods of linkage and methods of linkage quality evaluation should be provided. | Study design and data sources (page 8);  Supplement 1 (subsection 1) |
| **Results** | | | | | |
| Participants | 13 | (a) Report the numbers of individuals at each stage of the study (*e.g.*, numbers potentially eligible, examined for eligibility, confirmed eligible, included in the study, completing follow-up, and analysed)  (b) Give reasons for non-participation at each stage.  (c) Consider use of a flow diagram | Results section: 1st paragraph;  Figure 1.  Supplement 1: eFigure 2. | RECORD 13.1: Describe in detail the selection of the persons included in the study (*i.e.,* study population selection) including filtering based on data quality, data availability and linkage. The selection of included persons can be described in the text and/or by means of the study flow diagram. | Results section: 1st paragraph;  Figure 1.  Supplement 1: eFigure 2. |
| Descriptive data | 14 | (a) Give characteristics of study participants (*e.g.*, demographic, clinical, social) and information on exposures and potential confounders  (b) Indicate the number of participants with missing data for each variable of interest  (c) *Cohort study* - summarise follow-up time (*e.g.*, average and total amount) | Table 1;  Supplement 1: eTable 3; Subsection 5. |  | Table 1;  Supplement 1: eTable 3; Subsection 5. |
| Outcome data | 15 | *Cohort study* - Report numbers of outcome events or summary measures over time  *Case-control study* - Report numbers in each exposure category, or summary measures of exposure  *Cross-sectional study* - Report numbers of outcome events or summary measures | Table 1. |  | Table 1. |
| Main results | 16 | (a) Give unadjusted estimates and, if applicable, confounder-adjusted estimates and their precision (e.g., 95% confidence interval). Make clear which confounders were adjusted for and why they were included  (b) Report category boundaries when continuous variables were categorized  (c) If relevant, consider translating estimates of relative risk into absolute risk for a meaningful time period | Figure 2; Table 2  Supplement 1: eTables 7-9. |  | Figure 2; Table 2  Supplement 1: eTables 7-9. |
| Other analyses | 17 | Report other analyses done—e.g., analyses of subgroups and interactions, and sensitivity analyses | Table 2  Supplement 1: eTables 7-9. |  | Table 2  Supplement 1: eTables 7-9. |
| **Discussion** | | | | | |
| Key results | 18 | Summarise key results with reference to study objectives | Discussion section: 1^st^ paragraph |  | Discussion section: 1^st^ paragraph |
| Limitations | 19 | Discuss limitations of the study, taking into account sources of potential bias or imprecision. Discuss both direction and magnitude of any potential bias | Discussion section: Strengths and limitations | RECORD 19.1: Discuss the implications of using data that were not created or collected to answer the specific research question(s). Include discussion of misclassification bias, unmeasured confounding, missing data, and changing eligibility over time, as they pertain to the study being reported. | Discussion section: Strengths and limitations |
| Interpretation | 20 | Give a cautious overall interpretation of results considering objectives, limitations, multiplicity of analyses, results from similar studies, and other relevant evidence | Discussion section: Strengths and limitations |  | Discussion section: Strengths and limitations |
| Generalisability | 21 | Discuss the generalisability (external validity) of the study results | Strengths and limitations |  | Strengths and limitations |
| **Other Information** | | | | | |
| Funding | 22 | Give the source of funding and the role of the funders for the present study and, if applicable, for the original study on which the present article is based | Funding/support. |  | Funding/support. |
| Accessibility of protocol, raw data, and programming code |  | .. | Study Protocol (citation order in manuscript - 34). | RECORD 22.1: Authors should provide information on how to access any supplemental information such as the study protocol, raw data, or programming code. | Study Protocol (citation order in manuscript - 34). |

*Reference: Benchimol EI, Smeeth L, Guttmann A, Harron K, Moher D, Petersen I, Sørensen HT, von Elm E, Langan SM, the RECORD Working Committee. The REporting of studies Conducted using Observational Routinely-collected health Data (RECORD) Statement. *PLoS Medicine* 2015; in press.

*Checklist is protected under Creative Commons Attribution ([CC BY](http://creativecommons.org/licenses/by/4.0/)) license.
